# Proteomics and integrative omic approaches for understanding host–pathogen interactions and infectious diseases

**DOI:** 10.15252/msb.20167062

**Published:** 2017-03-27

**Authors:** Pierre M Jean Beltran, Joel D Federspiel, Xinlei Sheng, Ileana M Cristea

**Affiliations:** ^1^ Department of Molecular BiologyLewis Thomas LaboratoryPrinceton UniversityPrincetonNJUSA

**Keywords:** networks, omics, organelle, systems biology, virus infection, Genome-Scale & Integrative Biology, Microbiology, Virology & Host Pathogen Interaction, Post-translational Modifications, Proteolysis & Proteomics

## Abstract

Organisms are constantly exposed to microbial pathogens in their environments. When a pathogen meets its host, a series of intricate intracellular interactions shape the outcome of the infection. The understanding of these host–pathogen interactions is crucial for the development of treatments and preventive measures against infectious diseases. Over the past decade, proteomic approaches have become prime contributors to the discovery and understanding of host–pathogen interactions that represent anti‐ and pro‐pathogenic cellular responses. Here, we review these proteomic methods and their application to studying viral and bacterial intracellular pathogens. We examine approaches for defining spatial and temporal host–pathogen protein interactions upon infection of a host cell. Further expanding the understanding of proteome organization during an infection, we discuss methods that characterize the regulation of host and pathogen proteomes through alterations in protein abundance, localization, and post‐translational modifications. Finally, we highlight bioinformatic tools available for analyzing such proteomic datasets, as well as novel strategies for integrating proteomics with other omic tools, such as genomics, transcriptomics, and metabolomics, to obtain a systems‐level understanding of infectious diseases.

## Proteomics: a powerful tool for studying of infectious diseases

The constant interaction between hosts and pathogens is one of the most intriguing aspects of life. These interactions have been shaped throughout millions of years of evolution; hosts develop defense mechanisms against pathogenic invasions and pathogens circumvent these new lines of defense. Although adaptation processes have allowed hosts to co‐exists with and sometimes even benefit from pathogens, numerous pathogens are still etiological agents for a myriad of life‐threatening human diseases. Thus, understanding host–pathogen interactions has been a driver for the development of means to prevent and treat infection‐induced diseases.

**Figure 1:**
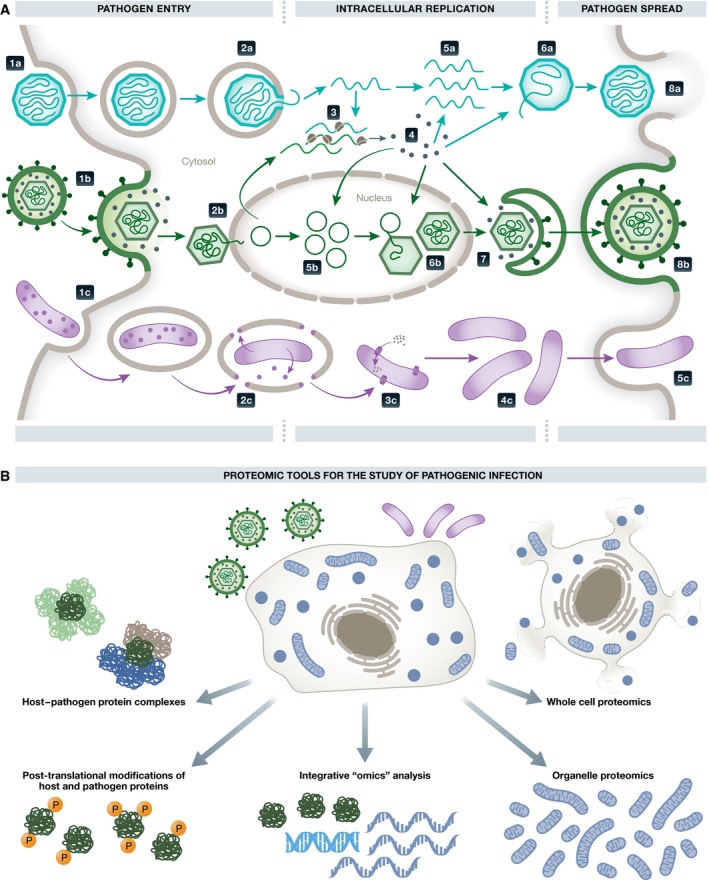
Proteomic tools used in the study of pathogenic infection (A) Overview of the replication cycle of intracellular pathogens. General steps are shown for cytosolic‐replicating non‐enveloped viruses (a), nuclear‐replicating enveloped viruses (b), and cytosolic bacteria (c). Viruses enter the cell through endocytosis (1a) or fusion with the host cell membrane (1b). The viral genome is extruded from the capsid to the cytosol (2a) or nucleoplasm (2b). Viral genes are then expressed (3) to produce viral proteins (4). Viral proteins facilitate immune evasion, viral genome replication (5a and 5b), viral genome encapsidation (6a and 6b), and envelopment (7). Fully assembled infectious viruses are secreted through lysis (8a) or through exocytosis (8b). Bacteria become endocytosed by the host cell (1c), followed by release from endocytic vesicles mediated by bacteria‐secreted proteins (2c). Then, the bacteria acquire nutrients directly from the host cytosol (3c). Bacteria replicate in the cytosol (4c) and exit the cell to the extracellular space or directly to neighboring cells (5c). (B) Overview of proteomic tools that have been utilized in the of study host–pathogen interactions.

At the molecular level, host–pathogen interactions occur regularly throughout the pathogen replication cycle. This is relevant for intracellular pathogens, such as viruses and cytosolic bacteria, which will be the focus of this review. Although their replication strategies vary, these pathogens need to accomplish several tasks in order to successfully replicate: enter the cell, harness cellular components (e.g., proteins, metabolites, lipids) for replication, and spread to neighboring cells (Fig 1A). Another important aspect of their replication cycle is the ability of pathogens to counteract host defenses, such as the immune system. Numerous years of research on the molecular biology of pathogen infections have established this general understanding of the pathogen “life cycle” within the host cell. However, several questions remain challenging and time‐consuming to address using classic molecular biology methods. Contemporary challenges include, first, the emergence of new pathogens and related infectious diseases, which demand the timely discovery of host and viral targets for diagnosis and development of therapeutics (Morens & Fauci, 2012; Malone *et al*, 2016). Second, the emergence of drug‐resistant viruses and bacteria highlights the need to discover host pathways that can be targeted to block the spread of pathogens. Lastly, medically threatening viruses that have been investigated for many years still persist with no suitable treatments or vaccines (e.g., Rieder & Steininger, 2014).

During the last decade, omic approaches have emerged as effective tools in basic, translational, and clinical research for the study of biological pathways involved in pathogen replication, host response, and disease progression. Proteomics, the study of the protein complement of biological systems, has become a driver in the discovery and understanding of host–pathogen interactions (Lum & Cristea, 2016; Fig 1B). This was made possible by both improved proteomic technologies, offering sensitive protein detection and quantification, and the increased awareness within the microbiology community, allowing the application of these approaches in innovative ways. The integration of proteomics with other biochemical and molecular biology methods, as well as with other omic approaches, has expanded the repertoire of tools to study pathogen infections. Genomic, transcriptomic, and metabolomic studies provide orthogonal information that complements proteomic analyses to achieve a systems‐level understanding of the infection process. Here, we review the current state of proteomics approaches to elucidate host–pathogen protein interaction networks, alterations in the composition and organization of the host cell proteome, and infection‐induced post‐translational regulation. We also discuss bioinformatic tools that form an integral part of proteomics to increase the power of discovery and ability to interpret large datasets. Finally, as datasets from other omic fields become available, we explore how multi‐omic technologies yield additional insight into the mechanics of host–pathogen interactions.

## Host–pathogen protein–protein interactions

**Figure 2:**
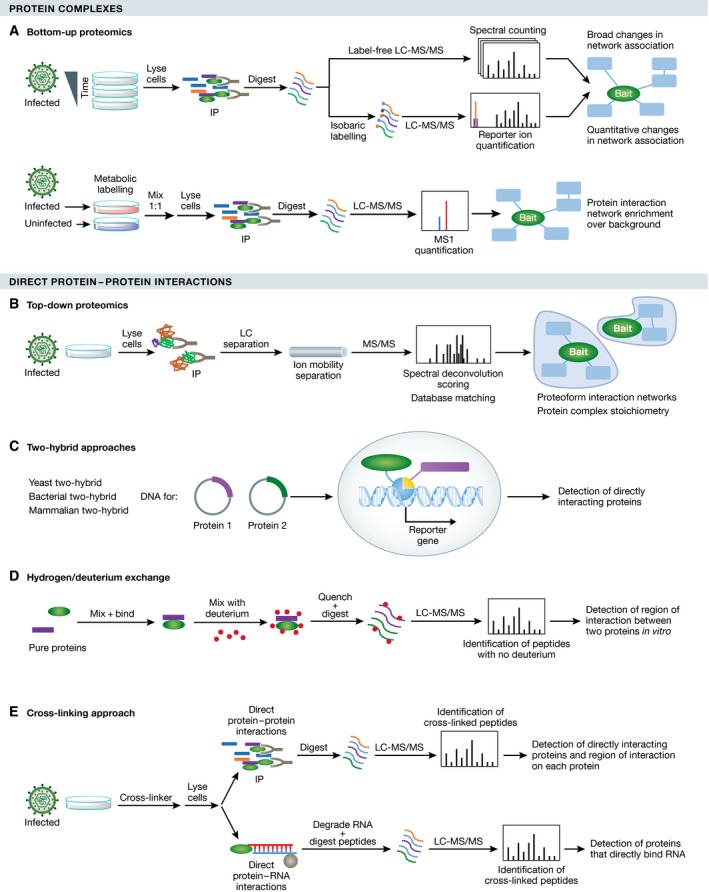
Proteomic tools to study protein–protein interactions in pathogenic infections (A) Detection of PPIs via shotgun IP‐MS. Quantitation can be done via label‐free (top), isobaric tagging (middle), or SILAC (bottom) strategies. (B) Detection of PPIs via top‐down MS. This method differentiates multiple intact different complexes containing the same protein of interest and can provide stoichiometry. (C) Detection of direct PPIs via Y2H. This method allows detection of direct interactions without the complexity of a cross‐linker, but at the expense of a high false‐positive rate and non‐infection contexts. (D) Characterization of PPIs by hydrogen/deuterium exchange. This method identifies the regions of each protein that interact *in vitro* and can be used to derive kinetic information about the interaction. (E) Detection of direct PPIs via cross‐linker. This method also identifies the regions of interaction on each protein and can be used in cells or *in vitro*.

Upon pathogen entry into a host cell, the progression of an infection relies on temporally and spatially regulated host–pathogen interactions that represent anti‐ and pro‐pathogenic cellular responses (Fig 1A). As intracellular pathogens must overcome host defenses and then reproduce in order to propagate, pathogen proteins interact with host proteins to either suppress or hijack the normal host protein functions (Lum & Cristea, 2016). Identification of these protein–protein interactions (PPIs) is not only critical for understanding the biology of infection, but can also point to novel targets in treatments against human pathogens. Beyond identification of a PPI, its characterization as either direct (one protein physically interacting with another) or indirect (proteins interacting via other intermediate molecules) can provide mechanistic insights into the host–pathogen interaction network. Here we review proteomics methods (Fig 2) that can be used to discover host–pathogen interaction networks, intact protein complexes, or direct interactions, and discuss their strengths, limitations, and future promising directions in the context of studying infectious diseases.

### Building host–pathogen protein interaction networks

The method that has seen the widest implementation in host–pathogen interaction studies is immunoaffinity purification coupled to mass spectrometry (IP‐MS) (Lum & Cristea, 2016). In IP‐MS, a protein of interest is isolated using either an antibody raised against the endogenous protein or by epitope‐tagging the protein of interest and using an antibody against that epitope. Then, the protein of interest and co‐isolated interacting proteins are identified by MS. When studying host–pathogen associations, advantages of IP‐MS are that experiments can be performed in relevant cellular model systems and in the context of viral infection to enable unbiased detection of PPIs, as reviewed in Greco *et al* (2014). These studies can be performed from the pathogen perspective, for example, isolating a viral protein to understand what host factors are targeted by the virus to ensure its replication or suppress host defense. Alternatively, IP‐MS studies can determine alterations in the interactions of a cellular protein during infection to characterize possible changes in the host protein functions. Given the temporal cascade of cellular events that occur during a pathogen infection (Fig 1A), IP‐MS methods, in conjunction with fluorescent tags and microscopy, were also designed to provide spatial–temporal information about host–pathogen interactions. Initially demonstrated for studying the RNA virus Sindbis (Cristea *et al*, 2006), this approach was later applied to other viruses, such as the RNA virus respiratory syncytial virus (Wu *et al*, 2012) and the DNA viruses human cytomegalovirus (HCMV) and pseudorabies virus (PRV) (Moorman *et al*, 2010; Kramer *et al*, 2012). During the past decade, IP‐MS approaches have provided a wide range of biological insights into the progression of an infection. For example, in investigating the prevalent human pathogen, HCMV, for which a vaccine or an effective antiviral treatment is still lacking, IP‐MS studies have led to the discovery of numerous mechanisms through which HCMV modulates cellular processes, for example, activation of the mTOR pathway to suppress host stress response (Moorman *et al*, 2008), inhibition of host sensing of viral DNA and immune signaling (Li *et al*, 2013), or use of cellular trafficking pathways during maturation of infectious particles (Moorman *et al*, 2010). The value of IP‐MS was also demonstrated for other viruses, for example, revealing that influenza A repurposes cellular nucleophosmin to aid viral RNA synthesis (Mayer *et al*, 2007) and that PRV uses a host kinesin‐3 motor for its trafficking in neurons (Kramer *et al*, 2012). Similarly, from the host perspective, IP‐MS has helped to define mechanisms of cellular defense (Diner *et al*, 2015) and to distinguish protein domain‐dependent interactions and functions for host antiviral factors (Diner *et al*, 2016).

Although IP‐MS has been successfully employed to study several viruses, there are still challenges associated with this method. The ability to tag a viral protein with an epitope for purification, while keeping the virus replication competent, can be problematic, particularly for viruses with smaller genome sizes. Because of this, several studies have utilized ectopic expression of tagged viral proteins outside the context of infection to acquire information of potential viral‐host PPIs that can be pursued with biological analyses. For example, this approach was shown valuable for studying the function of the Ebola virus matrix protein, VP40 (Yamayoshi *et al*, 2008). Another example is the interactome of all 18 human immunodeficiency virus (HIV) proteins, which predicted almost 500 host–pathogen interactions (Jager *et al*, 2012). Nevertheless, a continuous effort remains to study tagged proteins in the context of infection, such as a recent study using a scanning mutagenesis approach to generate replication‐competent HIV encoding tagged proteins for IP‐MS analyses (Luo *et al*, 2016).

In terms of quantifying the identified host–pathogen interactions, most IP‐MS studies have relied on label‐free MS quantification (e.g., spectral counting), which is simple, versatile, and can be applied to any biological system. However, labeling MS strategies provide more accurate quantification of PPI data and can be used to compare uninfected and infected samples in the same MS experiment (Fig 2A). The labeling can occur at the protein level, through the use of stable isotope labeling of amino acids in cell culture (SILAC), or at the peptide level, through incorporation of tandem mass tags (TMT) or other isobaric tags (Bantscheff *et al*, 2012). In studying host–virus interactions, SILAC was employed to control for false‐positive PPI identifications, such as when studying hepatitis C virus (HCV; Gerold *et al*, 2015). For bacteria, IP‐MS was used to identify interactions between effector proteins secreted by intracellular *Salmonella* and host proteins, and SILAC quantification helped assess specificity of interactions (Auweter *et al*, 2011). Although not yet used in host–pathogen PPI studies, the multiplexing capability of TMT (as many as 10 samples analyzed at once) would allow for the simultaneous quantification of multiple infection time points along with negative controls to evaluate the specificity of the interactions detected. Label‐free and isotopic labeling studies are not mutually exclusive, and several studies have combined SILAC with label‐free IP‐MS to great effect. For example, a combined analysis was used to determine both specific interactions of histone deacetylases by label‐free methods and the relative stability of these interactions by SILAC (Joshi *et al*, 2013). Such approaches can therefore be expanded to provide valuable information about dynamic host–pathogen interactions.

As indicated above, one limitation to IP‐MS datasets is the presence of non‐specifically interacting proteins that co‐purify with the protein of interest. One important consideration in pathogen–host interaction studies is that infections can trigger significant changes in protein abundances within a cell, and the background of non‐specific associations can be quite different than the one observed in an uninfected cell. Therefore, control isolations should be performed in the same biological context tested. Several available computer algorithms exist that use data from control and experimental isolations to help filter false‐positive PPIs (Armean *et al*, 2013). One such algorithm is the significance analysis of interactome (SAINT; Choi *et al*, 2011), which assigns interaction specificity scores to filter low‐confidence interactions. Informatics approaches can also be used to further refine identified interactions, for example, by providing additional controls for non‐specific associations, such as the contaminant repository for affinity purification (CRAPome; Mellacheruvu *et al*, 2013). A recent database for HSV‐1 interactions, HVint, provides an integrated resource of HSV‐1 protein interactions and further predicts additional interactions using evolutionary conservation of herpesvirus proteins (Ashford *et al*, 2016). Once a list of interactions is obtained, these PPIs are typically visualized within a functional network, which helps to identify the underlying biology in host–pathogen interactions. Common resources for network visualization include STRING (Szklarczyk *et al*, 2015) and Cytoscape (Cline *et al*, 2007), and we point the readers to a protocol guiding users through IP‐MS data analysis (Morris *et al*, 2014). Although the tools mentioned above have become fairly standard for the proteomics field, the virus–host PPIs for numerous viruses remain uncharted, and the understanding of their temporal and spatial regulation remains limited even for well studies viruses. IP‐MS has been employed to an even lesser extent in bacterial infections, and future studies are expected to continue to expand the use of quantitative proteomics in understanding infectious diseases.

### Analysis of intact protein complexes

In order to carry out different functions, proteins frequently exist simultaneously within distinct protein complexes. Therefore, although IP‐MS offers inventories of protein interactions, it averages together multiple protein complexes that contain the same protein of interest. Additionally, without further fractionation and analysis, information about the stoichiometry of associations within a complex is lost (only an average stoichiometry can be estimated). Top‐down MS analyses, in which proteins are analyzed without proteolytic digestion, can help obtain information about an intact protein or multiprotein complex (Toby *et al*, 2016; Fig 2B). When performed under non‐denaturing conditions, this technique can preserve both the non‐covalent interactions and the post‐translational state of the proteins within the complex. To date, in the context of infectious disease, this technique has been applied primarily to individual pathogen proteins, such as the hepatitis C virus pore protein p7 (Konijnenberg *et al*, 2015), and pathogenic complexes reconstituted *in vitro*, such as the Norwalk virus‐like particles (Shoemaker *et al*, 2010). However, top‐down MS has not been applied to studying host–pathogen complexes. The ability to analyze high molecular mass complexes remains challenging, but MS instrumentation improvements are steadily extending the mass range in which these analyses can be applied. Top‐down MS was also combined with ion mobility separation to learn about the overall shape of a multiprotein complex. A prime example comes from the Heck laboratory for the investigation of viral capsid nucleation and assembly for hepatitis B virus and norovirus (Uetrecht *et al*, 2011). Thus, top‐down MS is emerging as a promising tool for the study of host–pathogen protein complexes beyond the study of capsid assembly complexes.

### Detecting direct interactions

While the methods discussed above provide unbiased detection of interactions (IP‐MS) and information about the complex stoichiometry (top‐down MS), these approaches are not able to classify PPIs as direct or indirect. A classic method for detecting direct PPIs is the yeast two‐hybrid (Y2H) assay (Fig 2C), which was used to examine interactions between Epstein–Barr virus proteins and human proteins (Calderwood *et al*, 2007). Although not an intracellular pathogen, the *Enterohemorrhagic E. coli* (EHEC) has a close intracellular interaction with its host, as it injects at least 39 proteins into the host cytosol. Y2H was also used to elucidate direct PPIs between EHEC and the human host cells (Blasche *et al*, 2014). A downside for Y2H is its relatively high false‐positive rate due to the non‐physiological expression of proteins in cellular compartments in which they might not normally be expressed. Additionally, since pathogen proteins are expressed outside the context of an infection, many potentially relevant interactions can be missed. Another *in vitro* method used to identify the interacting regions of two proteins is hydrogen/deuterium exchange in conjunction with MS (Fig 2D). This technique was applied to study HIV assembly, identifying intermolecular interactions in immature and mature virion assembly complexes (Monroe *et al*, 2010).

The use of cross‐linking strategies has recently expanded the investigation of direct host–pathogen protein interactions (Fig 2E). For example, cross‐linking coupled with MS was used to identify epitopes to neutralizing antibodies in HCMV glycoproteins (Ciferri *et al*, 2015). This experiment was complemented with hydrogen/deuterium exchange to confirm peptides forming direct interactions. Advances in search algorithms designed for cross‐linking MS studies have improved their ease of use (Leitner *et al*, 2016). Additionally, a cross‐linking technology containing an affinity handle cleavable by the MS instrument improved the detection of cross‐linked peptides and was effective for studying virus–plant protein interactions and their surface topologies (Chavez *et al*, 2012; DeBlasio *et al*, 2015). In addition to the identification of direct PPIs, cross‐linkers can stabilize weaker or transient interactions, improving their identification, however usually at the expense of increasing non‐specific associations. A recent study took advantage of these cross‐linking tools and computational development (i.e., XLinkDB) to generate a large dataset of direct interactions between human lung cells and *Acinetobacter baumannii,* a subset of which were shown to be important in bacterial invasion (Schweppe *et al*, 2015).

The use of cross‐linkers is not limited to identifying PPIs during infection. Photo cross‐linking was used to capture RNA–protein interactions, providing both stoichiometric and structural information about the beginning of HIV viral genome packaging (Kenyon *et al*, 2015). Several studies used cross‐linking MS to identify host proteins that bind to viral RNA during polio (Lenarcic *et al*, 2013) and dengue (Viktorovskaya *et al*, 2016) virus infections. The host proteins identified in each study were unique to the respective viral infection, and subsequent knockdown studies demonstrated the necessity of these proteins for efficient viral processes. The examination of RNA–protein interactions by MS promises to further expand our understanding of post‐transcriptional regulation processes that may play important roles during pathogenic infection.

## Pathogen‐induced proteome alterations in time and space

The production, degradation, and spatial reorganization of proteins are central for the replication of pathogens. For instance, viral proteins are produced and shuttled to the appropriate compartments (e.g., nucleus or organelle membranes) for virion assembly (Tandon & Mocarski, 2012). In many cases, the pathogen triggers changes in the levels of specific host proteins needed for replication. The host also responds to the pathogen invasion through global alterations in the proteome organization, important for mounting effective defenses. For example, innate immune and stress responses to pathogenic invasion can trigger regulation of tens‐to‐hundreds of proteins (Janssens *et al*, 2014). Proteomic technologies have been developed and adapted to studying the temporally and spatially separated steps of an infection process (Fig 1). These studies have provided insights into the regulation of specific time points of infection and the necessary subcellular compartment reorganization.

### Temporal analysis of the infected cellular proteome

**Figure 3:**
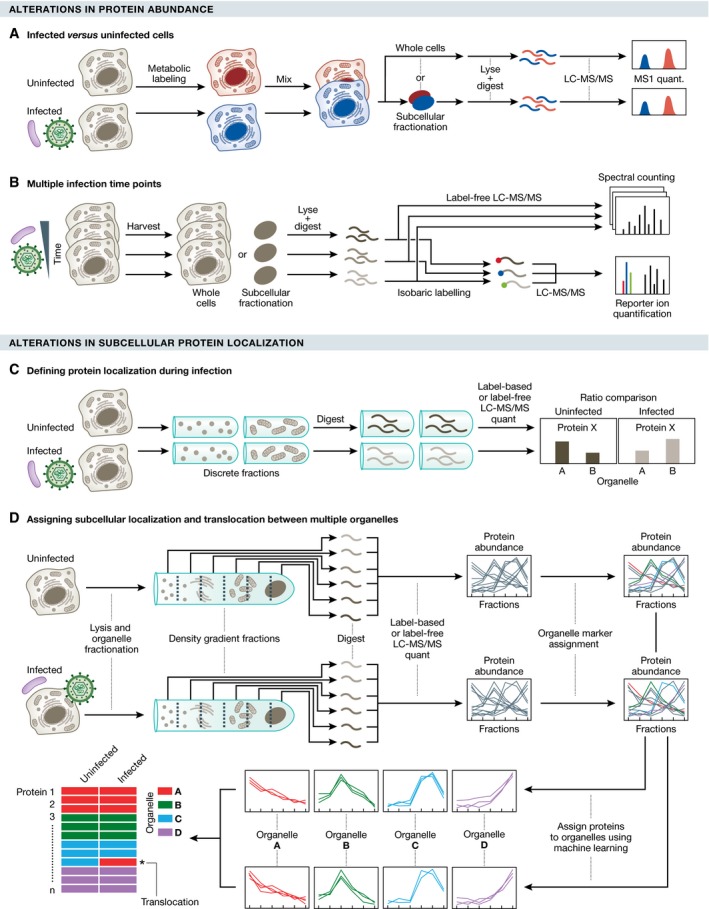
Proteomic tools to study whole‐cell or subcellular proteome alterations during infection Proteomic methods can be used to study alterations throughout infection in protein abundance or in protein subcellular localization. (A) SILAC‐based workflow to define proteome alterations upon infection. Differentially labeled uninfected and infected cells are mixed and processed directly for MS analysis (whole‐cell temporal proteomics) or preceded by a subcellular fractionation step (organelle temporal proteomics). (B) An alternative method using label‐free quantification or isobaric tags (e.g., TMT) to define proteome alterations at multiple time points of infection. Cells or subcellular organelles are harvested at different infection times, and following digestion, peptides from each fraction are analyzed by MS (label‐free quantification) or labeled with isobaric tags and mixed for multiplexed MS analysis and quantification. (C) Defining protein localization during infection. Discrete subcellular fractions (e.g., differential centrifugation or density gradient fractionation) are collected from infected and uninfected cells, and analyzed by MS. (D) Proteomic approach to assign proteins to specific organelles and determine alterations in protein subcellular localization. Multiple organelles from infected or uninfected cells are partially separated using a density gradient. Fractions are analyzed by quantitative MS, resulting in spatial profiles of proteins across the gradient. Well‐known organelle residents are used as organelle markers for the spatial profiles. The remaining proteins are assigned to organelles using classification algorithms (e.g., machine learning). The libraries of predicted protein localizations from infected and uninfected cells are compared to determine candidate proteins undergoing infection‐induced translocations between organelles.

Studying temporal proteome alterations has become a popular approach due to the availability of well‐established protocols and modern MS instrumentation. Experimental designs range from simple approaches, like comparing protein abundances between infected and uninfected cells (Fig 3A), to more complex approaches, such as including multiple time points (Fig 3B). For example, to study the replication cycle of herpesviruses, omic studies compared time points of infection that cover the infection life cycle in the cell (Weekes *et al*, 2014; Berard *et al*, 2015). Such studies implement label‐free and/or isotopic labeling methods (e.g., SILAC and TMT), in conjunction with analyses using private (Proteome Discoverer, Thermo Fisher) or open source software (Cox *et al*, 2014), to determine alterations in protein abundances. Once the relative quantitative values are collected, the significance and magnitude of the differential protein abundance are assessed with statistical methods, such as *t*‐test, analysis of variance (ANOVA), or more sophisticated linear models available in software packages (e.g., MSstats; Choi *et al*, 2014). Additional bioinformatics analyses are required to correlate the proteins with specific biological pathways and cellular functions. Commonly, hierarchical clustering is used to identify sets of proteins with similar temporal profiles upon infection. These clusters are then subject to functional analysis by a variety of bioinformatics tools, including gene ontology analysis, pathway analysis (Kanehisa *et al*, 2016), network analysis (Cline *et al*, 2007; Szklarczyk *et al*, 2015), or a combination of these (da Huang *et al*, 2009; Mi *et al*, 2013).

Temporal proteome analyses have been successful in identifying pathways regulated by the pathogen and key proteins involved in pathogenicity. For example, viruses depend on cellular metabolism and have acquired mechanisms to regulate it for energy production and lipid synthesis, among other processes. Broad alterations in proteins involved in metabolism regulation have been reported from temporal proteomic studies of human‐relevant viruses, such as the recently re‐emerged Chikungunya virus (Abere *et al*, 2012), HCMV (Weekes *et al*, 2014; Jean Beltran *et al*, 2016), flaviviruses (Pastorino *et al*, 2009; Grabowski *et al*, 2016), and HCV (Diamond *et al*, 2010). Some of these changes are temporally controlled; for example, HCV regulation of glycolysis proteins occurred only early in infection, while proteins used in lipid metabolism were increased throughout all time points (Diamond *et al*, 2010). These proteome alterations can also correlate with pathogenicity, as it was demonstrated in temporal proteomic studies of different influenza strains (Simon *et al*, 2015; Ding *et al*, 2016). Particularly, regulation of specific proteins by the emerging and highly virulent H7N9 influenza virus was associated with its increased cytopathic effects (Ding *et al*, 2016). Since infections induce broad proteome alterations, studies were also designed with a narrow focus on individual pathogenic proteins. For instance, a recent proteomic study expressing the RTA protein coded by Kaposi's sarcoma‐associated virus (KHSV), which triggers lytic reactivation, identified ARID3B as a host protein important to initiate lytic replication (Wood *et al*, 2016). So far mainly used for cell culture systems, temporal proteomic analyses during infection have been successfully applied for *in vivo* studies in animal models challenged with viruses and bacteria (Fraisier *et al*, 2014; Lopez *et al*, 2016; Shen *et al*, 2016).

To understand the protein kinetics (i.e., synthesis, turnover, and degradation) that lead to these changes in protein abundance, several proteomic tools have been developed and applied in pathogen research. Identification of newly synthesized proteins, possible through a “click chemistry” labeling strategy (Dieterich *et al*, 2007), led to the discovery of nascent proteins synthesized at axon terminals during infection by neurotropic viruses (Koyuncu *et al*, 2013). Changes in protein turnover rates of *Salmonella* (Wang *et al*, 2016) have been measured using dynamic SILAC (Claydon & Beynon, 2011) to identify how this pathogen regulates its proteome as it invades the host. Viruses can also trigger protein degradation using proteases encoded in their genome. Thus, the use of “degradomics” tools (i.e., the determination of protease substrates; Kleifeld *et al*, 2011) has revealed a novel target of the poliovirus 3C proteinase important for viral replication (Jagdeo *et al*, 2015).

### Spatial cellular proteome organization during infection

Although proteome analyses on whole cells reveal infection‐induced changes in protein abundances, it does not preserve spatial information that is important for understanding proteome organization and for characterizing molecular mechanisms of pathogen infection. As pathogens trigger alterations within specific subcellular compartments, several groups have used subcellular fractionation to investigate the localization of proteins and specific proteome alterations during infection. This method, referred to as spatial or organelle proteomics, is achieved by separating various subcellular compartments using fractionation methods, followed by MS analysis (Fig 3C). Fractionation methods include labeling and affinity purification (e.g., for cell surface proteins; Gudleski‐O'Regan *et al*, 2012), differential or density gradient centrifugation, and differential detergent fractionation. To quantify protein abundances in compartments of infected and uninfected cells, cells can be labeled using SILAC, combined, and fractionated, minimizing technical variability during the fractionation steps. Alternatively, the uninfected and infected samples can be kept separate during fractionation, and quantification can be performed by either label‐free approaches or isobaric tags (e.g., TMTs). The advantage of these alternatives is that there is less limitation in the number of samples that can be compared, allowing analysis of multiple fractions and infection time points.

These approaches have been applied to the targeted study of several organelles during infection. To better understand the requirements for viral entry into a host cell, and with the hope to design interventions that block this, infection‐induced changes in the cell surface proteome have been investigated. These studies demonstrated the dynamic role of the plasma membrane proteome in intracellular and intercellular signaling, transport of metabolites with the extracellular space, and cell attachment during infection (Gudleski‐O'Regan *et al*, 2012; Hsu *et al*, 2015; Matheson *et al*, 2015). The nucleus is a major target for nuclear‐replicating viruses such as herpesviruses, which target nuclear shuttling during their replication (Sanchez‐Quiles *et al*, 2011; Carter *et al*, 2015). The mitochondrion has crucial functions for viral replication, such as in cell metabolism, respiration, and stress response. Proteomic studies have shown viral‐induced alterations in the mitochondria biogenesis, oxidative phosphorylation, and the electron transport chain (Wu *et al*, 2013; Karniely *et al*, 2016; Villeneuve *et al*, 2016). Suborganellar compartments, such as the endoplasmic reticulum mitochondria‐associated membranes (MAMs), have also been investigated, as these are sites of innate immune signaling through MAVS (Horner *et al*, 2015). Secretory organelles are involved in the replication of certain pathogens, such as *Salmonella typhimurium*, and are amenable targets for proteomic analyses to identify host factors required for replication (Kaloyanova *et al*, 2015).

It is starting to become clear that viruses can cause alterations in multiple compartments during infection. Understanding the temporal correlation of these changes and the possible translocation of proteins between organelles demands the simultaneous analysis of multiple subcellular compartments throughout an infection time course. Methods, such as PCP (protein correlation profiling) and LOPIT (localization of organelle proteins by isotope tagging), can generate protein localization maps of multiple organelles by matching the fractionation patterns of organelle markers with other organelle proteins separated by density gradient fractionation (Andersen *et al*, 2003; Dunkley *et al*, 2004). An extension of these methods was recently developed to define the dynamic spatial organization of the host proteome across multiple organelles throughout the time course of HCMV infection (Fig 3D). Integrating quantitative proteomics with live cell microscopy, this study revealed broad alterations in organelle composition and shape, and identified discrete protein translocations between secretory organelles that were necessary for the production of infectious particles (Jean Beltran *et al*, 2016). In looking ahead, the integration of methods developed to track the dynamic localization of proteins within the cell (Itzhak *et al*, 2016) will also be valuable in studying the spatial reorganization of the cell proteome during infection.

## Pathogen‐induced regulation of protein post‐translational modifications

Post‐translational modifications (PTMs) regulate protein functions through alterations in protein interactions, stability, activity, and subcellular localization. Therefore, PTM regulation on either host or pathogens proteins can play critical roles in the progression and outcome of infection (Ribet & Cossart, 2010). Studying the cellular landscape of PTMs and their pathogen‐induced regulation has provided key insights into our understanding of host–pathogen interactions. This section will review PTMs studied in the context of infection, which have included phosphorylation, acetylation, glycosylation, ubiquitination, and SUMOylation.

### Diverse forms of post‐translational modifications are relevant in the context of infection

**Figure 4:**
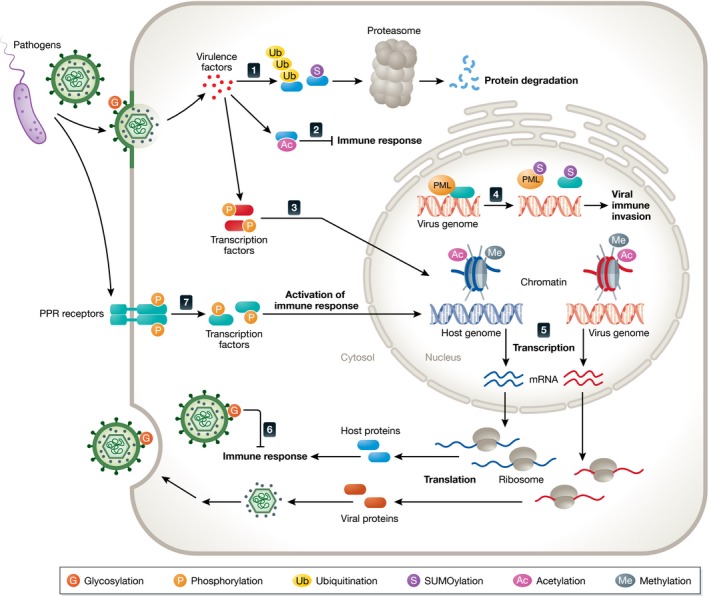
Post‐translational modifications (PTMs) involved in the context of infection Infections induce a series of dynamic PTMs on host and pathogen proteins that act in pro‐ and anti‐pathogen responses. (1) Virulence factors promote ubiquitination and SUMOylation of host proteins, resulting in degradation by the proteasome. (2) Acetylation is used by pathogens to block immune response. (3) Pathogen invasion causes phosphorylation of transcription factors, altering host and pathogen gene expression. (4) Viral genome can evade immune response by disrupting nuclear bodies via SUMOylation of PML and other nuclear body proteins. (5) Histones, finely‐tuned by PTMs, bind to host and viral genomes, modulating host and viral gene expression. (6) Viral glycoproteins on envelope are modified by glycosylation, facilitating viral entry and dampening immune response. (7) Pathogen infection activates PPR receptors, which relay the signal to transcription factors via adaptors to induce immune responses.

Post‐translational modifications are relevant throughout various stages of the pathogen life cycle (Fig 4). During entry, enveloped viruses fuse with the cellular membrane via viral glycoproteins on the viral envelope. Extensive glycosylation has been observed in envelope proteins of herpesviruses, including HSV‐1, HCMV, varicella zoster virus (VZV), and Epstein–Barr virus (EBV), and shown to be critical for their virulence (Bagdonaite *et al*, 2016). For instance, HCMV glycoproteins gB and gH/gL are essential for virus attachment to cell membrane and the subsequent membrane fusion (Wille *et al*, 2013). In addition to facilitating entry, highly glycosylated envelope proteins can protect both RNA and DNA viruses from detection by the host immune system (Aguilar *et al*, 2006; Reynard *et al*, 2009; Helle *et al*, 2010; Machiels *et al*, 2011). Interestingly, a similar mechanism of immune evasion has been observed in bacteria. The opportunistic pathogen *Burkholderia cenocepacia* shields the flagellar protein FliC from recognition by the host TLR5 receptor during membrane attachment via glycosylation, thus dampening the host immune responses (Hanuszkiewicz *et al*, 2014). Once inside the host cells, pathogens have strategies to regulate host protein PTMs to obstruct early host defenses. For instance, the HIV‐1 protein Vpu interferes with the ubiquitination machinery, inhibiting the degradation of IκBα (Bour *et al*, 2001). The bacterial pathogen *Yersinia enterocolitica* also targets this pathway by expressing the virulence factor YopJ/P that mediates acetylation of the IKK complex, dampening its activity, and blocking IκBα phosphorylation (Fig 4; Mittal *et al*, 2006). PTMs are also critical for regulating different steps in the virus life cycle. For example, HSV‐1 encodes ICP0, an E3 ubiquitin ligase, which mediates degradation of a class of host proteins via ubiquitination and SUMOylation, protecting viral genome from repression (Fig 4; Randow & Lehner, 2009). Additionally, phosphorylation of the human T‐cell lymphotropic virus (HTLV‐1) protein Tax was shown to activate viral gene transcription (Bex *et al*, 1999).

From the host perspective, PTMs can promote defense mechanisms against infection. PTMs play critical roles in pathogen recognition via host pattern recognition receptors (PRR) and their downstream innate immune pathways (Fig 4). For example, subcellular localization of IFI16, a cellular protein able to recognize viral DNA and induce innate immune response, is modulated by acetylation of its nuclear localization signal motifs (Li *et al*, 2012b). SUMOylation contributes to the stability of another PRR, cGAS, promoting sensing of DNA virus infection (Hu *et al*, 2016). Phosphorylation of innate immune adaptor proteins (e.g., MAVS, STING, and TRIF) activates IRF3, triggering the production of type I IFN (Liu *et al*, 2015). This phosphorylation‐mediated mechanism is present in distinct pattern recognition pathways (Fig 4), highlighting the importance of this modification in host–pathogen interactions [reviewed by Janeway and Medzhitov (2002)]. Finally, the NF‐κB pathway, a core innate immunity pathway, can be jointly regulated by multiple PTMs, including phosphorylation, ubiquitination, and acetylation (Viatour *et al*, 2005; Li *et al*, 2012a; Collins *et al*, 2016).

Another relevant PTM regulation during infection is the dynamic modification of histones, which alters chromatin compaction and the recruitment of histone modifiers. As cellular histones contribute to the formation of host nucleosomes, as well as viral nucleosome (e.g., nuclear‐replicating DNA viruses and integrating viruses), histone PTMs have significant impact on both host and pathogen gene expression. For example, an increasing body of evidence has shown that methylation and acetylation histone patterns are altered upon infection, which results in changes in chromatin remodeling and repression of immune factors [Fig 4; reviewed by Paschos and Allday (2010)]. However, histone PTM status is also critical for progression through the virus life cycle, as shown for the expression of immediate early, early, and late viral proteins upon infection with HCMV (Cuevas‐Bennett & Shenk, 2008). Another example comes from studies on HIV‐1, where host histone deacetylase 1 (HDAC1) was shown to reduce histone acetylation, impacting chromatin structure of HIV LTR and repressing viral transcription (Williams *et al*, 2006).

### MS as a tool to study host and pathogen protein PTMs

Post‐translational modifications are ubiquitous in the cell, and many of these are dynamically regulated during infection, motivating global PTM analyses made possible by proteomic methods. Selected global PTM mapping, that is, focused on specific types of modifications, has been performed for various pathogenic agents, including bacteria, fungi, protozoa, and viruses (e.g., Leach & Brown, 2012; Bell *et al*, 2013; Champasa *et al*, 2013; Liu *et al*, 2014; Bagdonaite *et al*, 2016) to identify and quantify SUMOylations, phosphorylations, acetylations, and histone modifications (Kulej *et al*, 2015). The primary means of PTM discovery experiments is the selective enrichment of specific proteins or PTMs followed by identification of modified peptides. This enrichment is usually accomplished via antibodies against the PTM or protein or via a resin that can enrich a class of PTMs based on their chemical properties (e.g., metal affinity chromatography for phosphopeptides) (Gruhler *et al*, 2005; Zielinska *et al*, 2012; Udeshi *et al*, 2013; Hendriks *et al*, 2014; Engholm‐Keller & Larsen, 2016). In addition to these discovery‐driven experiments, targeted MS/MS methods, such as selected reaction monitoring (SRM) or parallel reaction monitoring (PRM), provide the means for sensitive monitoring of PTMs on proteins of interest. Despite their well‐recognized value for the accurate quantification of low abundance PTMs (Kusebauch *et al*, 2016), these approaches have not been frequently implemented in pathogen infection studies, and their future use promises to expand our understanding of proteome regulation during infection. Additionally, the systematic investigation of different types of PTMs and of their temporal and spatial regulation in infectious contexts is still lacking. This is the case for PTMs that are known to be critical regulators of protein function, such as phosphorylation, ubiquitination, and acetylation, as well as for emerging PTMs with limited knowledge about their impact on protein function, such as malonylation, succinylation, and lipoylation. Furthermore, for the identified PTMs, the exact roles of many of these modifications either in uninfected or infected cells still remain to be elucidated. Future studies that combine whole proteome monitoring, protein–protein interaction studies, and PTM identification within a single biological source (e.g., relevant cells or tissue) will enhance our understanding of infection at a systems level and will help pinpoint new targets for therapeutic intervention.

## Multi‐omics integration for the study of host–pathogen interactions

**Figure 5:**
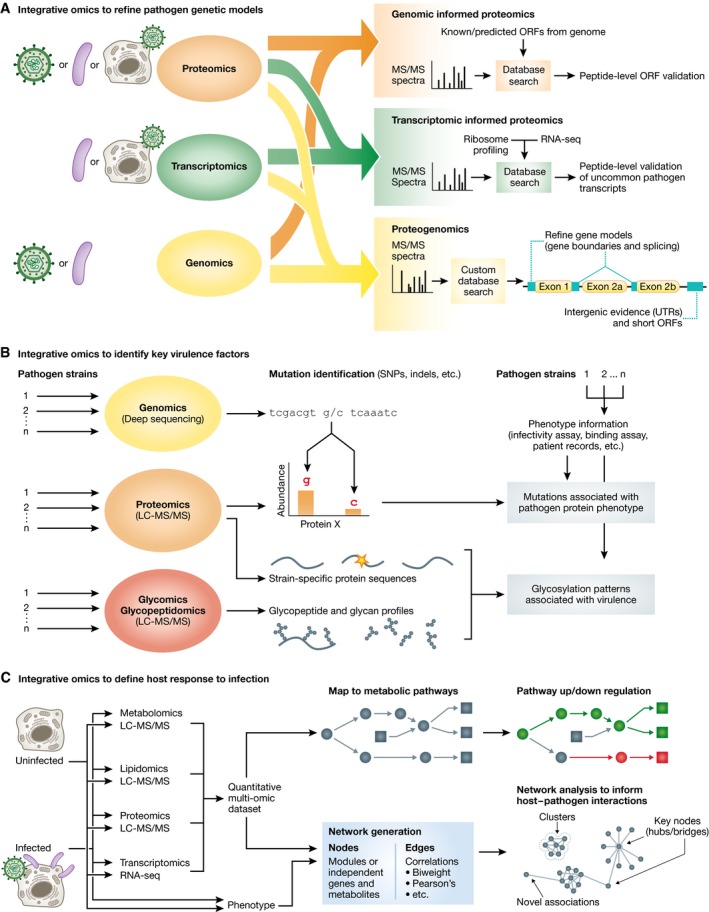
Integrative omic approaches for the study of host–pathogen interactions (A) Proteomic tools have been integrated with other omic approaches to refine models of pathogen genomes. Proteomic data can be acquired from purified virions, bacteria culture under host physiological conditions, and from infected host cells. Transcriptomic data are acquired from bacteria cultures or virus‐infected host cells. Genomic data are acquired from purified viral particles and bacteria cultures. In genomic‐informed proteomic experiments, MS data are searched through a genome database containing translated sequences from known or predicted open reading frames (ORFs) to provide evidence of protein expression. In transcriptomic‐informed proteomic experiments, the search database is built from RNA‐seq and ribosome profiling to identify uncommon transcripts difficult to predict from genomic sequences. Proteogenomic approaches use both genomic and transcriptomic data to build a customized database search useful in identifying peptides from 5′ untranslated regions (UTRs), 3′ UTRs, alternative junction splicing, intron sequences, short ORFs, and alternative reading frames to refine pathogen gene models. (B) Integrating proteomics with other omic tools and phenotype information to identify key virulence factors. Genomic data are used to identify strain‐specific mutations and associate them with differences in protein expression. The differences in protein expression are then related to phenotype alterations using phenotypic assays on wild‐type strains and those harboring the mutations. Proteomics, glycomics, and glycopeptidomics are used to identify glycosylation patterns associated with specific protein sequences and viral strains. These glycosylation patterns are then associated with phenotypic variations using phenotypic assays on strain having different glycosylation patterns. (C) Using multi‐omic datasets to define the host response to an infection. Quantitative multi‐omic datasets are mapped to known metabolic networks to identify pathways that are up‐ or downregulated upon infection. Alternatively, multi‐omic datasets are integrated with phenotype data to construct correlation networks. Nodes represent individual genes, metabolites or phenotype measurements, or they can also represent module eigengenes. Edges represent correlations between nodes from quantitative measurements. Networks can be analyzed to identify novel associations, including novel clusters, and key nodes, such as hubs and bridges.

Early omic approaches were mostly focused on acquiring a single “layer” of information from the cell as a whole. However, it is evident that pathogenic invasion, as well as other biological processes, causes alterations at multiple layers in the molecular organization of the host. Furthermore, these molecular layers (e.g., genetic sequence, mRNA transcripts, protein, lipids, metabolites) are interconnected, influencing and complementing each other. In recent years, a number of multi‐omic approaches have been applied to studying pathogenic infection by integrating proteomics with other omic analyses. These approaches have been useful to determine the coding capacity of pathogens, identify key virulence factors, and define, at a systems level, the response of the host to pathogenic infection (Fig 5).

One such multi‐omic approach is proteogenomics (Fig 5A), the integration of proteomic, transcriptomic, and genomic data to identify novel peptides and refine existing gene models (Nesvizhskii, 2014). Proteomic experiments informed by transcriptomic data (e.g., RNA‐seq and ribosome profiling) are of particular interest for the study of pathogens. This is because certain pathogens encode complex protein isoforms generated by non‐canonical translation events, overlapping and short open reading frames (ORFs), and complex alternative splicing and transcription start/end sites. Thus, defining genes based on genetic sequences and *in silico* approaches is not sufficient. One example is the HCMV genome, which was initially thought to encode ~192 unique ORFs by an *in silico* approach (Murphy *et al*, 2003), yet the coding capacity was revealed to be more complex using ribosome profiling (Stern‐Ginossar *et al*, 2012). Protein evidence of these non‐canonical ORFs has been collected by MS in the original ribosome profiling study and in following proteomic studies (Weekes *et al*, 2014; Jean Beltran *et al*, 2016). Conversely, proteomics is also integrated with transcriptomic analyses to improve the annotation of pathogen genomes, providing experimental evidence for genes, delineating intergenic events, and refining the boundaries of existing gene models of pathogens (Abd‐Alla *et al*, 2016; Miranda‐CasoLuengo *et al*, 2016). Although the data analysis on this types of experiments is challenging, computational platforms are readily available, which facilitate future proteogenomic research in pathogens (Fan *et al*, 2015; Rost *et al*, 2016).

Multi‐omic approaches have been adapted to identify key virulence factors (Fig 5B). Genetic factors (i.e., SNPs, non‐synonymous mutations, and genome rearrangement) that contribute to virulence and pathogenicity can be identified by sequencing and comparing genomes of multiple pathogen strains, as done in mycoplasma (Lluch‐Senar *et al*, 2015). In this study, additional transcriptomic and proteomic data were used to determine the mechanism underlying the genetic‐virulence relation. Elevated CARDS toxin expression was identified as a source of pathogenicity associated with a single nucleotide mutation specific to one mycoplasma strain. One source of virulence that is difficult to assess from genetic sequences or gene expression is the glycosylation pattern of pathogenic glycoproteins, such as the hemagglutinin receptors of influenza. Proteomics, glycopeptidomics, and glycomics were integrated to identify glycosylation sites and glycoform distribution among several influenza strains (Khatri *et al*, 2016). Using this approach, it was possible to determined that the glycosylation patterns correlated with selective pressure imposed by host immune factors (i.e., immune lectins), which affect the strain antigenicity and virulence.

Multi‐omic studies are also highly effective to analyze the response and alterations occurring in the host system (Fig 5C). Since pathogens commonly cause alterations in the host metabolism (Munger *et al*, 2008), several multi‐omic approaches have integrated proteomics and metabolomics to obtain a systems‐level understanding of metabolic pathway regulation upon infection (Su *et al*, 2014; Villar *et al*, 2015). In these studies, the added protein‐level information in metabolic pathways is used to identify specific proteins that may be targeted by pathogens to cause these metabolic alterations. To integrate multi‐omics data, network approaches (Bensimon *et al*, 2012) can explain the relation between different omic layers of information. By analyzing network topology, one can identify functional relations between nodes in the network and key regulators of a system. In an early example of multi‐omic network analysis during infection, proteomic and lipidomics data during HCV infection were used to generate a network relating proteins and lipids through abundance correlations (Diamond *et al*, 2010). The analysis revealed phospholipids and lipid‐regulating enzymes that act as hubs and are linked to HCV pathogenesis. The value of integrating multi‐omics datasets is also highlighted by the integrative personal omics profile (iPOP) analysis, which collected multi‐omic data of an individual throughout a 14‐month period that included two events of viral infection (Chen *et al*, 2012). Other studies have integrated phenotypic data from infections with multi‐omics data using network models (Gibbs *et al*, 2014). An exemplary study by (Tisoncik‐Go *et al*, 2016) investigated host responses during infection by various strains of pandemic influenza virus. Proteomics, lipidomics, metabolomics, and transcriptomics datasets were represented as module eigengenes (i.e., representative profiles of modules from each dataset using the first principal component) to generate an integrative network, identifying relationships between omic datasets and infection‐induced phenotypes. Furthermore, this study revealed strain‐specific multi‐omic associations by looking at the effects of these influenza strains separately.

As novel omics methods continue to be developed, their integration with other omics approaches will provide additional levels of information that could benefit pathogenic research. Some of these approaches could include, for example, integration of host and pathogen PTMs (Chen *et al*, 2016) or subcellular location information (Jean Beltran *et al*, 2016). An important aspect of multi‐omics studies is the availability of informatics platforms that can be used to access and visualize the data. Some examples include ImmuNet (Gorenshteyn *et al*, 2015), a tool that integrates immune pathway knowledge and omics data, and ZikaVR (Gupta *et al*, 2016), a tool for visualization of multi‐omic data from Zika virus research. The availability of such resources is at the core of generating data‐driven hypotheses for future pathogenic research.

## Closing remarks

To establish the interplay between host cells and pathogens, it is critical to gain a view of host and pathogen proteins on a systemic level. In recent years, the study of human infectious diseases has benefited significantly from the contribution of proteomic approaches for pathogenic research, and proteomic tools are now starting to become promising tools in clinical studies and diagnosis (Box 1). Quantitative MS‐based proteomic methods (e.g., TMT‐labeling and SILAC) have become well established for sensitive and accurate comparison of protein abundances between biological samples, and have been readily integrated for studying the temporal progression of an infection (Fig 1). Notably, a growing interest in this field is the understanding of these temporal alterations with spatial detail (i.e., within organelles, nucleus, or viral assemblies). Improvements in technologies, such as fractionation, sample multiplexing, and computational tools for spatial proteome analysis, are expected to facilitate proteomic research of infectious disease with both temporal and subcellular detail. A major component of understanding pathogen‐induced changes in proteome and related cellular pathways is the identification of PPIs between host and pathogen proteins and their dynamic regulation during the time course of infection. Future studies of such PPIs should leverage the existing proteomic platforms, while also taking advantage of the continually improving MS quantitative approaches, particularly for adding spatial and temporal resolution to the study of interactomes. Adding additional layers of complexity, PTMs have emerged as critical modulators of protein functions during infection. Proteomic studies on PTMs hold great potential for uncovering mechanisms mediating the progression, spread, and pathogenicity of infection.

**Box 1. Clinical applications of proteomic tools**

In addition to providing fundamental insights into the basic molecular mechanisms of infectious disease, proteomics has made a significant impact in clinical studies and diagnosis. The utility of proteomics as a clinical tool is perhaps best demonstrated in the area of cancer treatment. One example is the development of diagnostic tools for classification of malignant ovarian cancer, such as OVA1 (Fung, 2010) currently approved by the US Food and Drug Administration (FDA). Significant research progress has been made in the use of proteomics to inform about the need for a treatment. It is expected that further applications of proteomics will allow microorganism identification, biomarker discovery, and tracking the course of disease.

**Box 1 Figure:**
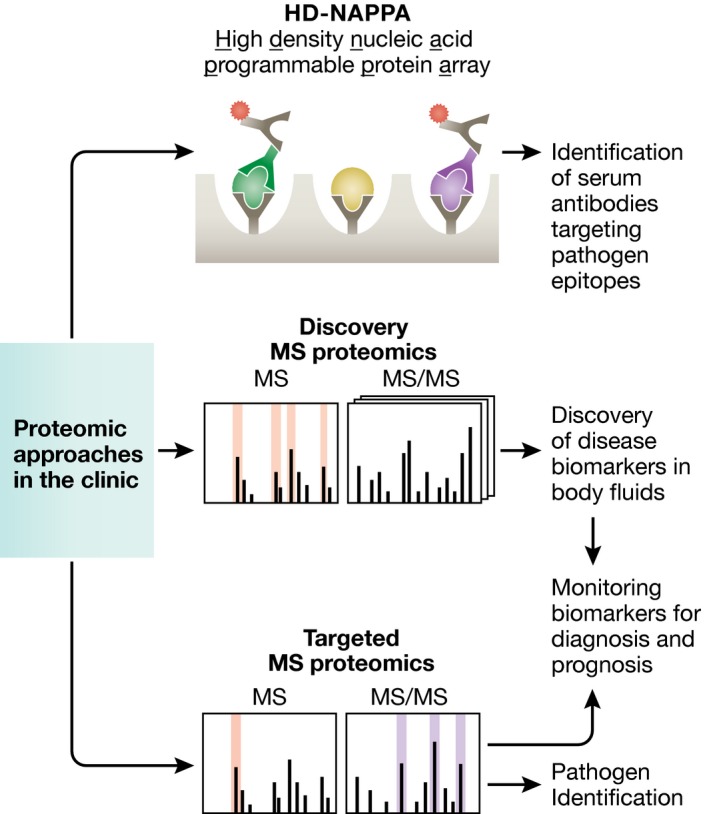
**Proteomic approaches in the clinic directed toward infectious diseases.** Diagram showing proteomic approaches that have been applied for the discovery, diagnosis, and prognosis of pathogens and infectious diseases. (Top) Protein arrays (e.g., HD‐NAPPA) exposing different viral proteins. If an antibody targeting these viral proteins is present in the serum of the individual, it binds and is detected using fluorescent secondary antibodies. (Middle) Using shotgun MS approaches, proteins can be readily detected in the patient body fluids. Significantly enriched proteins in diseased individuals are candidates as biomarkers. (Bottom) These biomarkers are then monitored using a targeted MS approach in diseased patients for diagnosis and prognosis. Additionally, a targeted MS approach can be used to readily identify pathogens from patient samples.

One of the crucial points for treating infectious diseases is the reliable and rapid identification of the microorganism causing the disease. One current limitation of standard diagnostic methods is the turnaround for laboratory cultures, which may take 2 days or more to complete analyses. Currently, two MALDI (matrix‐assisted laser desorption/ionization)‐MS instruments are approved by the US FDA for identification of cultured bacteria from human specimens, the VITEK MS (bioMerieux, Inc.) and the MALDI Biotyper CA System (Bruker Daltonics, Inc.; Bizzini *et al*, 2010; Rychert *et al*, 2013). Although these instruments were approved for use in small amounts of cultured bacteria, they do not circumvent the need to wait for the culture to grow. Several studies have demonstrated the effective detection of bacteria directly from human biological fluids, such as blood and urine (La Scola & Raoult, 2009; Inigo *et al*, 2016). Thus, it is expected that MS‐based proteomics will be used in the future as a tool for rapid identification of pathogens from human biological specimens.

In addition to identifying the pathogenic microorganism, proteomics techniques have been used to identify host biomarkers of infection. For example, MS has been used to identify host biomarkers for sepsis from urine samples (Su *et al*, 2013) and biomarkers associated with hepatocellular carcinoma in hepatitis B virus patients (Yau *et al*, 2013), which can become cost‐effective and non‐invasive diagnostic tools. Biomarkers are not only useful in diagnosis, but also in tracking the progress of a treatment. For example, MS was used to monitor the response to hepatitis C treatment to prevent unnecessary toxicities and reduce cost (Devitt *et al*, 2011). Similar monitoring of treatment progress could also be beneficial in clinical trials, such as for tuberculosis treatment (Nahid *et al*, 2014). Importantly, biomarkers can be readily screened by targeted MS/MS approaches (Chambers *et al*, 2014) for the development of clinical diagnosis protocols.

Finally, pathogens can trigger release of antibodies that can be identified to facilitate diagnosis and treatment of infection and some autoimmune disorders. An innovative proteomic tool for detection of these antibodies is the high‐density nucleic acid programmable protein array (HD‐NAPPA). These arrays express hundreds of viral proteins from multiple viral species, which can be used to detect antibodies in the human serum specific to viral epitopes (Bian *et al*, 2015). Thus, both MS‐based and non‐MS‐based technologies pave a promising future for the application of proteomics in clinical diagnosis and treatment.

Continued development of algorithms for analysis and interpretation of data from protein abundance, interactions, and PTMs will facilitate examination of the biology underlying pathogenic infection. Notably, there is a need for bioinformatics platforms for multi‐omics analyses, as laboratories that do not specialize in omics research, yet have particular expertise in pathogenic research, would benefit from implementing these approaches to their specific infectious disease models. Altogether, these proteomic studies have contributed to discoveries in diverse pathogenic infections, by characterizing pathogenic factors, host anti‐pathogen proteins and protein complexes, and profiling both host and pathogen PTM sites during infection. The integration of proteomics with other omic technologies provides researchers with opportunities to obtain a holistic picture of host–pathogen interaction, with the goal to obtain a better understanding of the dynamics of disease and for future discovery of therapeutic targets.

## Conflict of interest

The authors declare that they have no conflict of interest.
